# Comment on “Evaluation of the Long-Term Administration of Proton Pump Inhibitors (PPIs) in the Mineral Nutrient’s Bioavailability”

**DOI:** 10.1021/acsomega.6c04754

**Published:** 2026-05-20

**Authors:** Lourianne N. Cavalcante, Marcellus H. L. Ponte Souza

**Affiliations:** † School of Medicine, 28111 Federal University of Bahia, Reitor Miguel Calmon Av. S/N, Canela, Salvador 40110-905, Brazil; ‡ Department of Physiology and Pharmacology, Federal University of Ceará, Fortaleza 60020-181, Brazil

## Abstract

Proton pump inhibitors (PPIs), including omeprazole, are widely prescribed for acid-related disorders, with well-established efficacy and safety. A recent rat study published in *ACS Omega* ( de BritoA. S.,  ACS Omega 2025, 10, 56085−56095, 10.1021/acsomega.5c07700 41322596 PMC12658647) reported small hematological and mineral changes after 60-day omeprazole exposure and was publicly interpreted as evidence that PPIs cause anemia, bone disease, or cancer in humans. This Comment argues that such extrapolation is not supported by the data. Key methodological limitations include a subtherapeutic dose (0.68 mg/kg/day) for rodents, the absence of pharmacodynamic confirmation (gastric pH, H^+^/K^+^-ATPase activity), and a lack of specific markers for anemia (ferritin, transferrin, hepcidin), bone density, or functional mineral absorption. Substantial interspecies differences in gastric physiology, metabolism, and PPI pharmacokinetics preclude direct translation to humans. Meta-analyses involving millions of participants show a modest fracture risk, heavily confounded by age, frailty, and corticosteroid co-use, with no causal link to bone loss. Iron and vitamin B12 deficiencies, when present, are usually modest, multifactorial, and correctable; hypomagnesemia is rare. The historic gastric cancer signal reflected untreated *Helicobacter pylori*, not PPI toxicity. The genuine clinical concern is inappropriate, indication-free chronic PPI use, not an intrinsic hazard of omeprazole demonstrated in rodents.

The use of proton pump inhibitors (PPIs), such as omeprazole, is widespread in clinical practice for the management of acid-related disorders. Despite their efficacy and well-established safety profile, discussions regarding potential adverse effects associated with long-term use have gained prominence. Recently, an experimental study conducted in rats and published in *ACS Omega* (10.1021/acsomega.5c07700) was interpreted by its authors and disseminated by some media outlets as direct evidence that omeprazole could cause anemia, impair bone health, or increase cancer risk in humans. A careful examination of the study, however, indicates that such conclusions are not supported by the data presented.

The study evaluated hematological parameters and mineral distribution in the organs of rats exposed to omeprazole for up to 60 days. Small reductions in hemoglobin, discrete alterations in iron and calcium, and modest changes in leukocyte counts were observed. These findings are compatible with physiological effects of acid suppression in rodents, but they do not constitute disease or represent clinical outcomes. Furthermore, important methodological aspects limit the extrapolation of these results to humans.

First, the dose used (0.68 mg/kg/day) is lower than the doses typically required to produce significant acid suppression in rodents, which metabolize PPIs more rapidly than humans. The study also did not assess essential pharmacodynamic parameters, such as gastric pH or H^+^/K^+^-ATPase activity, making it impossible to confirm whether effective inhibition of acid secretion occurred. The absence of specific markers of anemia (ferritin, transferrin, hepcidin), bone density assessment, or functional measures of mineral absorption further restricts the interpretation of the findings.

Animal models are fundamental tools for investigating biological mechanisms, but substantial physiological differences between species prevent direct extrapolation. Rats have a higher basal gastric pH, accelerated metabolism, distinct pathways for the absorption of iron, calcium, magnesium, and vitamin B12, and different pharmacokinetics for PPIs. Without allometric dose equivalence and pharmacodynamic confirmation, transposing these results to humans becomes inappropriate.

The clinical literature on PPIs and bone health is extensive and more robust than any isolated animal study. Meta-analyses involving millions of participants show a modest increase in relative fracture risk, with considerable heterogeneity and strong influence from confounding factors such as advanced age, baseline frailty, and concomitant corticosteroid use.^1,2^ Cohort studies adjusted for comorbidities frequently do not demonstrate a significant association. There is no evidence that PPIs directly cause bone loss.

The hypothesis that PPIs could induce iron-deficiency anemia stems from the reduction in gastric acidity, which contributes to the solubilization of nonheme iron. Human studies show that long-term use may be associated with a discrete reduction in iron stores, particularly in older adults, but overt anemia is uncommon and generally multifactorial.^3^ Clinical trials do not demonstrate significant decreases in hemoglobin in healthy adults.

Regarding vitamin B12, the risk of deficiency associated with prolonged PPI use is documented but modest, and it is more evident in older adults and after years of continuous therapy.^4–6^ Supplementation readily corrects the deficiency when present. PPI-induced hypomagnesemia, although recognized, is rare, occurs after prolonged use, and is associated with individual predisposition, polypharmacy, or renal disease.^7,8^

Finally, there is no evidence that PPIs cause cancer in humans. Initial associations with gastric cancer were later attributed to untreated *Helicobacter pylori* infection rather than the medication itself.^9^ International guidelines reinforce that PPIs are safe when used according to appropriate clinical indications.^10,11^

The central issue, therefore, is not the inherent risk of omeprazole but the inappropriate and prolonged use without formal indication, a common practice in several countries. Indiscriminate use may mask underlying diseases, hinder diagnosis, and expose vulnerable patients to avoidable adverse effects. An evidence-based approach recommends that PPIs be used at the correct dose, for the appropriate duration, and with clinical monitoring.

The Brazilian experimental study (10.1021/acsomega.5c07700) contributes to the mechanistic understanding of PPI effects but does not provide evidence that omeprazole causes anemia, bone loss, or cancer in humans. Responsible interpretation of the data requires acknowledging the limitations inherent to animal models and the need for clinical studies to assess actual risks in the population.
